# *Escherichia coli* outer membrane vesicles can contribute to sepsis induced cardiac dysfunction

**DOI:** 10.1038/s41598-017-16363-9

**Published:** 2017-12-12

**Authors:** Kristina Svennerholm, Kyong-Su Park, Johannes Wikström, Cecilia Lässer, Rossella Crescitelli, Ganesh V. Shelke, Su Chul Jang, Shintaro Suzuki, Elga Bandeira, Charlotta S. Olofsson, Jan Lötvall

**Affiliations:** 1 Department of anesthesiology and Intensive Care Medicine, Institute of Clinical Science, Sahlgrenska Academy, University of Gothenburg, Gothenburg, 40530 Sweden; 2 Krefting Research Centre, Institute of Medicine, University of Gothenburg, Gothenburg, 40530 Sweden; 3 Bioscience, Cardiovascular and Metabolic Diseases, IMED Biotech Unit, AstraZeneca, Mölndal, 43150 Sweden; 4 Codiak BioSciences Inc, 500 Technology Square, 9th floor, Cambridge, MA 02139 USA; 5 Department of Physiology/Metabolic Physiology, Institute of Neuroscience and Physiology, Sahlgrenska Academy, University of Gothenburg, Gothenburg, 40530 Sweden

## Abstract

Sepsis induced cardiac dysfunction (SIC) is a severe complication to sepsis which significantly worsens patient outcomes. It is known that bacteria have the capacity to release outer membrane vesicles (OMVs), which are nano-sized bilayered vesicles composed of lipids and proteins, that can induce a fatal inflammatory response. The aim of this study was to determine whether OMVs from a uropathogenic *Escherichia coli* strain can induce cardiac dysfunction, and to elucidate any mechanisms involved. OMVs induced irregular Ca^2+^ oscillations with a decreased frequency in cardiomyocytes through recordings of intracellular Ca^2+^ dynamics. Mice were intraperitoneally injected with bacteria-free OMVs, which resulted in increased concentration of pro-inflammatory cytokine levels in blood. Cytokines were increased in heart lysates, and OMVs could be detected in the heart after OMVs injection. Troponin T was significantly increased in blood, and echocardiography showed increased heart wall thickness as well as increased heart rate. This study shows that *E. coli* OMVs induce cardiac injury *in vitro* and *in vivo*, in the absence of bacteria, and may be a causative microbial signal in SIC. The role of OMVs in clinical disease warrant further studies, as bacterial OMVs in addition to live bacteria may be good therapeutic targets to control sepsis.

## Introduction

Sepsis represents a syndrome defined as life-threatening organ dysfunction caused by a dysregulated host response to infection^1^. The incidence of sepsis is increasing globally, and is a major cause of death, affecting all age groups^1,2^. Septic-induced cardiomyopathy (SIC) is a severe and common complication of septic shock, present in more than 40% of cases, and is associated with increased morbidity and mortality^2,3^. Extensive research over decades suggest that SIC has a complex pathogenesis, and multiple pathways may be involved^2,4^.

Both eukaryotic and prokaryotic cells release extracellular vesicles, which are known to mediate intercellular communication^5,6^. Gram-negative bacteria release outer membrane vesicles (OMVs), which carry many virulent factors, including cell free bacterial peptidoglycan and lipopolysaccharide^7,8^. OMVs can contribute to pathogenicity in infection by carrying and transmitting virulence factors and act as a mechanism for colonization^9,10^. It has been shown that OMVs may initiate a sepsis-like inflammatory response, and activate platelets, immune- and endothelial cells to further induce endothelial disruption and a procoagulant state^11–14^. The effects of OMVs on the heart has previously not been studied.

We here hypothesize that OMVs can induce SIC, and that any such effect may be direct on cardiomyocytes. To study this, we isolated OMVs from a uropathogenic *Escherichia coli* (*E. coli)* strain and applied these OMVs to cardiomyocytes *in vitro*, as well as in mice *in vivo*, quantifying cell or organ function, as well as inflammatory responses.

## Results

### Isolation and characterization of *E. coli*-derived OMVs

**Figure 1:**
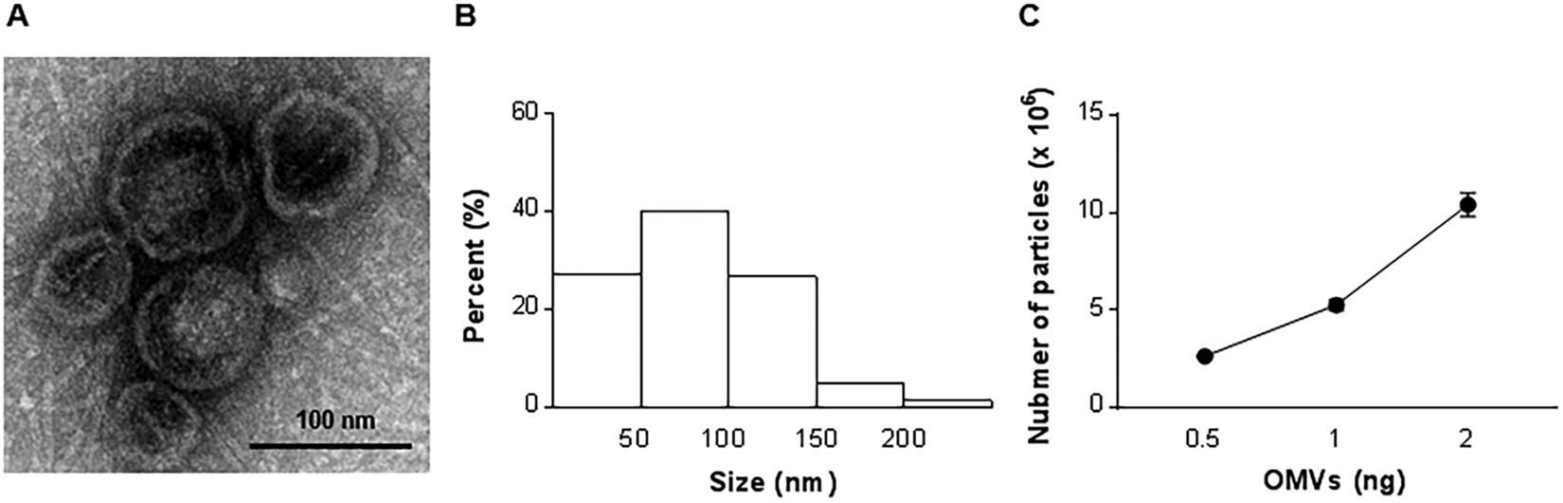
Characterization of *E. coli*-derived OMVs. (**A**) Transmission electron microscopy image of purified OMVs. Scale bar, 200 nm. (**B**) Size distribution of OMVs according to diameter. The size total 1189 OMVs were determined by using the IMOD program. (**C**) The OMVs particle number per OMVs proteins measured by nanoparticle tracking analysis.

OMVs from *E. coli* were visualized by transmission electron microscopy **(**TEM) (Fig. 1A), and appeared as spherical structures with an average diameter of 87.5 ± 7.8 nm (Fig. 1B). Nanoparticle tracking analysis revealed that the ratio of particles to ng of OMVs proteins was 5.3 ± 0.5 million (Fig. 1C). The OMV isolation completely lacked whole bacteria, as no bacterial structures were observed in TEM, and attempts to culture bacteria from the OMV isolates in Luria-Bertani broth failed to result in bacterial growth (Supplementary Fig. 1). By extracting LPS from OMVs, it was calculated that the OMVs consist of 60 ng (60.0%) of LPS per 100 ng of OMVs proteins (data now shown).

### Cellular uptake, cell viability and inflammatory responses of OMVs in HL-1 cells

**Figure 2:**
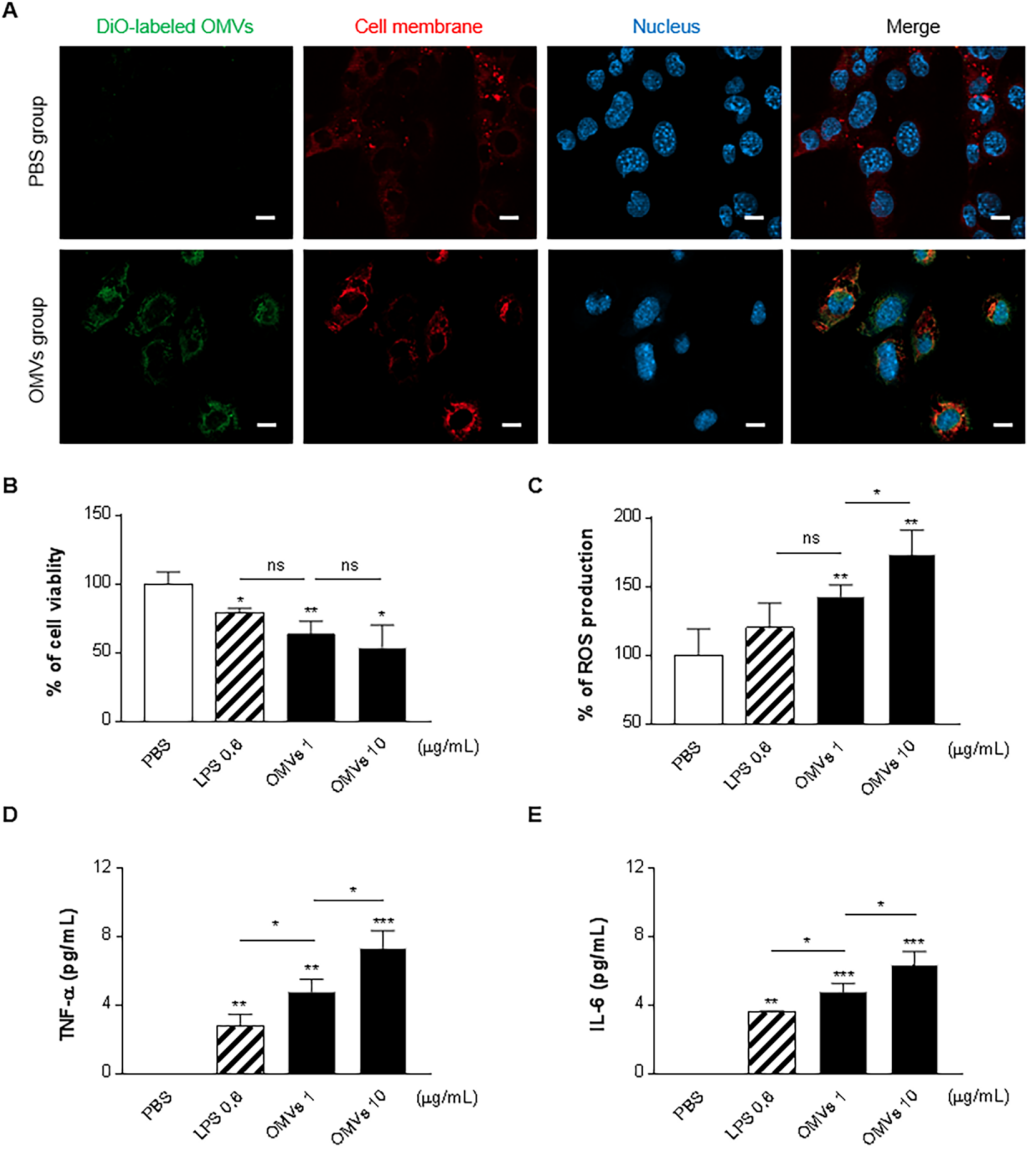
OMVs in HL-1 induce cytotoxicity and pro-inflammatory cytokine production. (**A**) OMVs (10 μg/mL) were incubated with cells for 6 h. OMVs, cell membrane, and nuclei were stained by DiO (Green), Cellmask Deep Red (Red), and DAPI, respectively. Scale bars, 20 µm. (**B**) Cells were exposed to LPS (0.6 µg/mL) or OMVs (1, 10 µg/mL) for 48 h and cell viability was assessed by MTT assay. Results are expressed in percentage of control. (**C**) Cell were treated with the peroxide-sensitive florescent probe 2′,7′-dichlorofluorescein di-acetate, followed by incubation with LPS (0.6 µg/mL) or OMVs (1, 10 µg/mL) for 3 h. Fluorescence was read at 560 nm, and ROS generation was expressed as percent change compared to the untreated control. (**D** and **E**) LPS (0.6 µg/mL) or OMVs (1, 10 µg/mL) were added to the cells, and the culture supernatant concentrations of TNF-α and IL-6 24 h later are shown in panel **D** and **E**, respectively. *P < 0.05; **P < 0.01; ***P < 0.001; ns, not significant; versus PBS group. LPS (0.6 µg/mL) and OMVs (10 µg/mL) group were also compared with OMVs (1 µg/mL) group. Error bars indicate SEM. n = 3.

HL-1 cells were stained with the membrane-specific stain Cellmask Deep Red before incubation with DiO-labeled OMVs. As shown in the confocal micrographs in Fig. 2A, OMVs were associated to the cell membranes, but could also be observed inside of cells. Moreover, dynosore, an endocytosis inhibitor, totally blocked entry of the OMVs into HL-1 cells (Supplementary Fig. 2).

A MTT assay revealed that OMVs reduce viability in HL-1 cells (Fig. 2B). OMVs cause decreased viability of cells at both 1 and 10 µg/mL concentrations, without evident dose-dependency. The effects of LPS 0.6 µg/mL (corresponding to the amount of LPS included in 1 µg/mL of OMVs) induced similar reduction in cell viability as the OMVs (1 µg/mL). Compared to phosphate-buffered saline (PBS), OMVs caused a dose-dependent increase in intracellular reactive oxygen species **(**ROS) accumulation, whereas the LPS did not (Fig. 2C).

To determine whether the isolated OMVs induce cytokine release from cardiomyocytes and macrophages, TNF-α and IL-6 were measured in the supernatants of HL-1 (Fig. 2D,E) and RAW 264.7 cells (Supplementary Fig. 3). OMVs provoked a significant production of TNF-α and IL-6 in both HL-1 and RAW 264.7 cell, both being significantly greater than the effects of LPS alone, suggesting that other OMV components contribute to this cellular response. To investigate if the response of HL-1 cells to OMVs or LPS is merely related to cell death, hydrogen peroxide as another cell-killing agent and non-lethal concentration of OMVs and LPS were tested in HL-1 cells (Supplementary Fig. 4A). Hydrogen peroxide did not show the production of pro-inflammatory cytokines, but even low and non-lethal concentration of OMVs and LPS could induce a significant increase in cytokines (Supplementary Fig. 4B,C), revealing that the results obtained above are not simply due to large-scale cell death.

We next examined if this cytokine induction accompanies to nuclear factor-ĸB (NF-ĸB) activation, following NF-ĸB p65 subunit phosphorylation. Western blot analysis for phosphorylated p65 protein levels revealed that both OMVs and LPS induce phosphorylation of this molecule to a similar level, although the used concentration of OMVs was 10% of that of LPS (Supplementary Fig. 5), indicating that OMVs likely represent a more potent effect in cytokine mediated p65 phosphorylation, implicating activation of NF-ĸB signaling.

To investigate if there is difference between commercial LPS used in this study and LPS in the OMVs, LPS banding patterns were compared, showing that both of them have similar molecular size of the lipid A, but O antigen structure is slightly different (Supplementary Fig. 6A). However, commercial LPS and LPS from OMVs could induce a significant increase in pro-inflammatory cytokines in HL-1 cells to a similar extent (Supplementary Fig. 6B), revealing that both LPS have similar inflammatory activity despite different O antigen structure.

### Effects of OMVs on the intracellular calcium concentration in HL-1 cells

**Figure 3:**
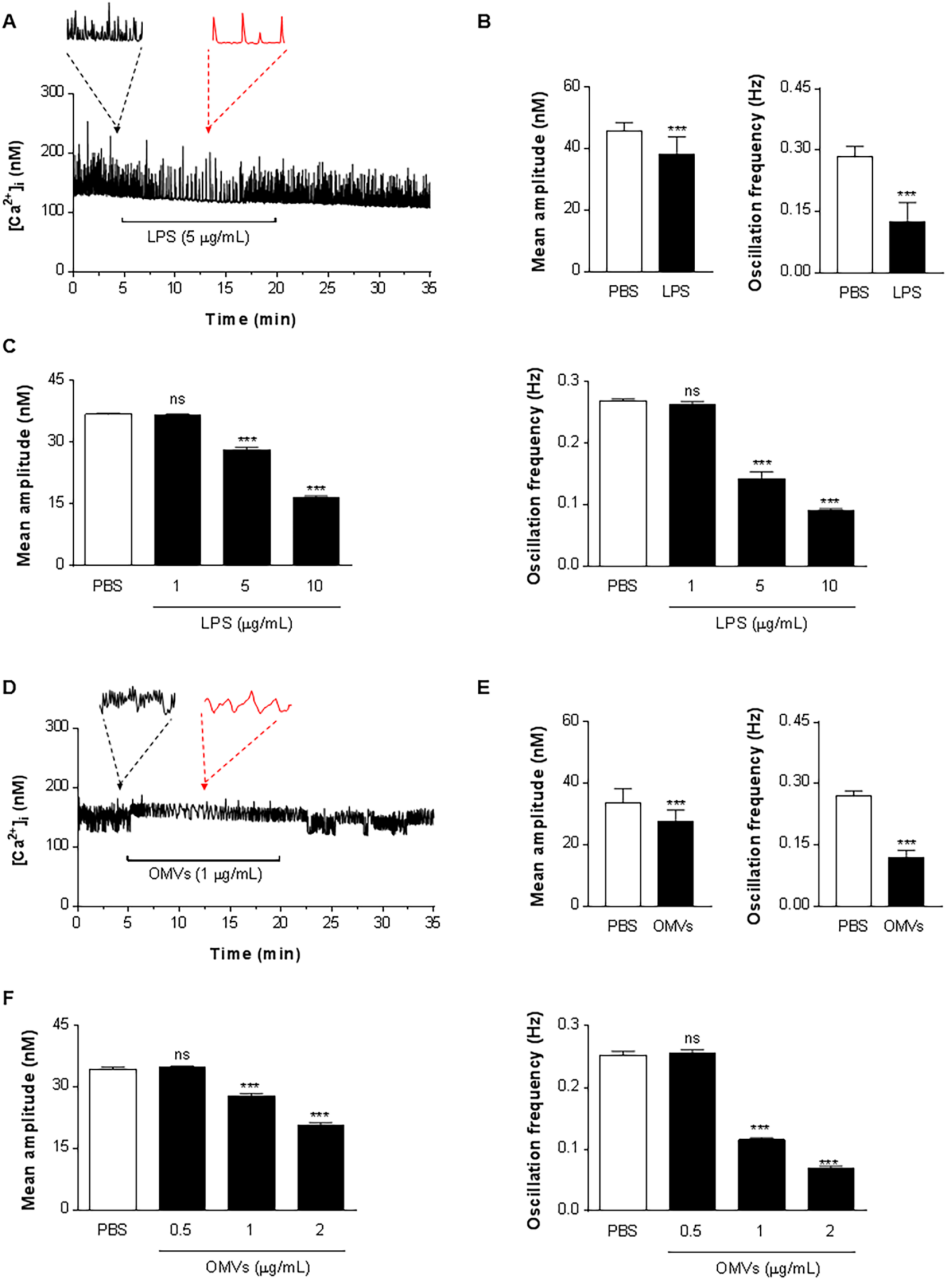
OMVs affect the change of intracellular calcium concentration in HL-1 cells. (**A** and **D**) LPS (5 µg/mL) (**A**) or OMVs (1 µg/mL) (**D**) was applied for 15 min to the cells which were continuously perfused with the extracellular solution. Horizontal lines show application time of LPS or OMVs. Black and red arrows/dashed lines indicate the expanded traces acquired at a time point prior to (4 min) and following (14 min) infusion of LPS or OMVs. Traces show representative recordings from four separate experiments. (**B** and **E**) Mean amplitude and oscillation frequency of LPS- (**B**) and OMVs-treated cells (**E**) were quantified out of a total of 30 analyzed ROIs from four separate experiments in each group. (**C** and **F**) HL-1 cells were treated with LPS (1, 5, 10 µg/mL) (**C**) or OMVs (0.5, 1, 2 µg/mL) (**F**) for 15 min, and then mean amplitude and oscillation frequency were quantified out of a total of 30 analyzed ROI from four separate experiments in each group. ***P < 0.001; ns, not significant; versus PBS group. Error bars indicate SEM.

As calcium is a critical regulator of cardiomyocyte function, especially contractility^15^, changes in intracellular calcium following OMVs exposure was studied. Considering that LPS has been implicated in endotoxin-mediated cardiac dysfunction, we also determined whether LPS directly influence cardiomyocyte cytosolic Ca^2+^ ([Ca^2+^]_i_). In agreement with previous studies^16^, fura-2 loaded HL-1 cells displayed rhythmic [Ca^2+^]_i_ oscillations. Perfusion of HL-1 cells with LPS (5 µg/mL) caused a decrease in amplitude and frequency of [Ca^2+^]_i_ oscillations (Fig. 3A,B). Subsequently, washout of LPS resulted in recovery of [Ca^2+^]_i_ oscillations. The effects of OMVs on HL-1 cell [Ca^2+^]_i_ oscillations was studied using various doses of OMVs (0.5, 1 and 2 µg/mL). The minimum concentration of OMVs required to affect amplitude and frequency of [Ca^2+^]_i_ oscillations was 1 µg/mL (Fig. 3F), which is 20% of the effective minimum concentration of LPS (5 µg/mL; Fig. 3C). An LPS concentrations of 1 µg/mL was without effect on [Ca^2+^]_i_ oscillations (Fig. 3C). As shown in Fig. 3D,E, and Supplementary Fig. 7, OMVs (1 µg/mL) induce reduced amplitude and frequency of [Ca^2+^]_i_ oscillations similar to LPS (5 µg/mL). Interestingly, [Ca^2+^]_i_ oscillations were not completely recovered after OMV washout (Fig. 3D and Supplementary Fig. 7), in contrast to LPS washout, arguing that OMVs have a more prolonged and potent activity despite lower concentration than LPS.

### Effects of polymyxin B on OMVs-induced calcium change

**Figure 4:**
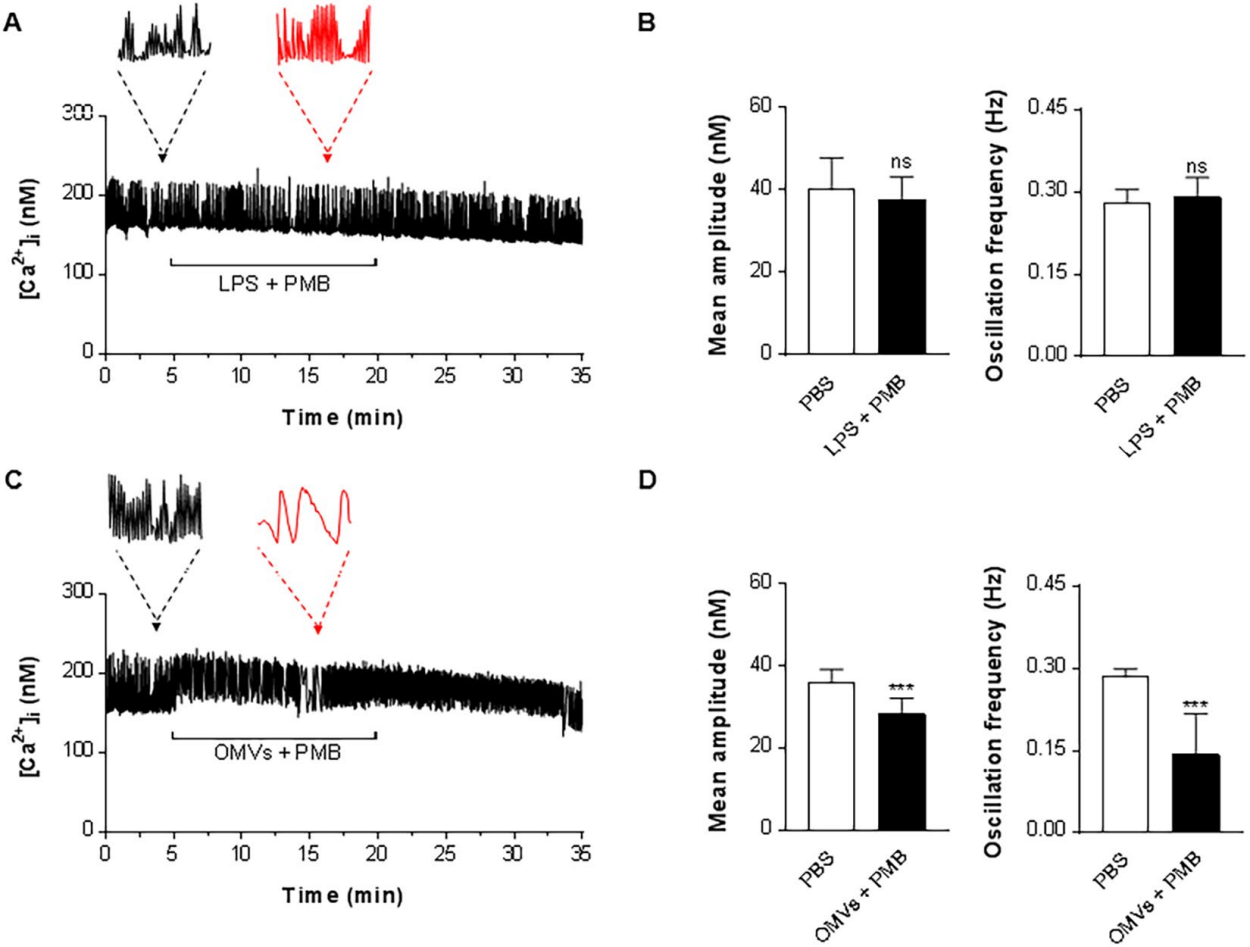
Both vesicular LPS and other components contribute to OMVs-induced contractility dysfunction. (**A** and **C**) LPS (5 µg/mL) (**A**) or OMVs (1 μg/mL) (**C**) were pre-incubated with polymycin B (PMB; 5 µg/mL) for 10 min, where after cells were treated with the mixture by addition to the extracellular perfusion solution. Horizontal lines show application time of LPS + PMB or OMVs + PMB. Black and red arrows/dashed lines indicate the expanded traces acquired at a time point prior to (4 min) and following (16 min) infusion of LPS or OMVs. Traces show representative recordings s from four separate experiments in each group. (**B** and **D**) Mean amplitude and oscillation frequency of LPS- (**B**) and OMVs-treated cells (**D**) were quantified out of a total of 30 analyzed ROI from four separate experiments in each group. ***P < 0.001; ns, not significant; versus PBS group. Error bars indicate SEM.

Polymyxin B, which is known to inhibit LPS, was used to separate the effect of the LPS component in OMVs for the effects in [Ca^2+^]_i_ oscillations. We first confirmed that polymyxin B perfusion did not affect [Ca^2+^]_i_ oscillation amplitude or frequency (data not shown). When LPS, pre-treated with polymyxin B, was added to HL-1 cells, no alterations of [Ca^2+^]_i_ oscillation amplitude or frequency were observed as shown in Fig. 4A,B. When OMVs, pre-treated with polymyxin B were added to HL-1 cells, the amplitude and frequency of [Ca^2+^]_i_ oscillations were in contrast still reduced (Fig. 4C,D), to a similar degree as the caused by OMVs without polymyxin B (Fig. 3D).

### OMVs-induced cardiac injury and inflammation in mice

**Figure 5:**
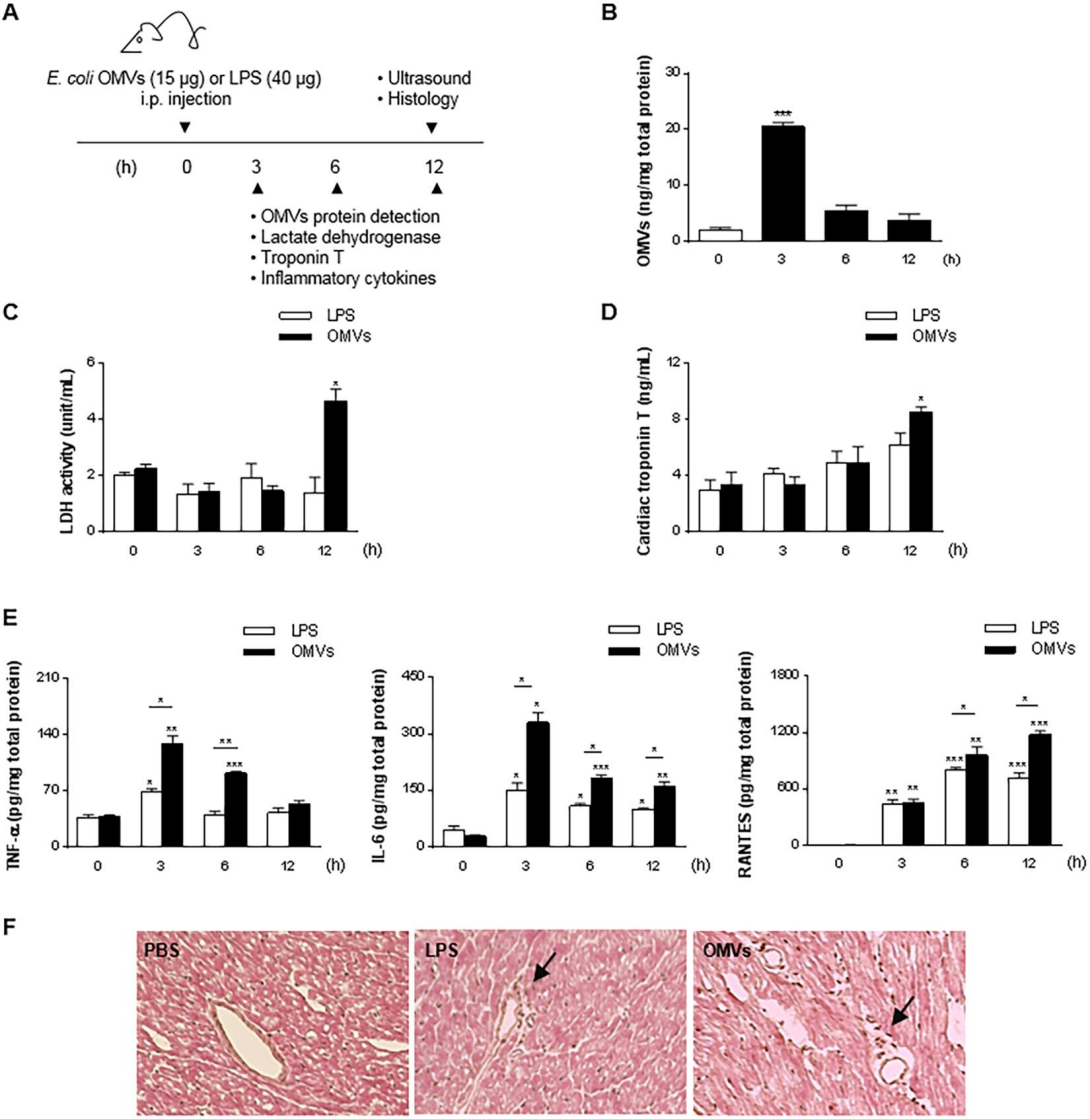
OMVs provoke cardiac injury and inflammation *in vivo*. (**A**) Study protocol for investigation of inflammation in heart. OMVs (15 µg) or LPS (40 µg) was injected i.p once, and then mice were sacrificed at 3, 6, and 12 h. (**B**) OMVs proteins were quantified in heart lysates using anti-OMVs antibody. (**C** and **D**) Lactate dehydrogenase **(**LDH) (**C**) activity and Troponin T (**D**) levels were quantified in serum. (**E**) Inflammatory cytokines (TNF-α, IL-6, and RANTES) in heart lysates (**F**) Representative hematoxylin and eosin stained section of hearts at 12 h following administration of OMVs or LPS. Original magnification: 200x. *P < 0.05; **P < 0.01; ***P < 0.001; versus 0 h group. Error bars indicate SEM. n = 5.

To verify the *in vitro* result suggesting that OMVs induce cardiomyocyte injury and inflammation, mice were injected intraperitoneally (i.p) once with PBS, OMVs (15 µg) or LPS (40 µg, which is calculated as more than 4-fold more LPS than in 15 µg of OMVs) followed by analysis at 3, 6, and 12 h, as shown in Fig. 5A. OMVs treated animals showed eye exudates and reduction in motility, while there was no change in phenotype for LPS and PBS treated animals (data not shown). The amount of OMV proteins in the heart tissue was quantified by using an anti-OMVs antibody, which detects outer membrane proteins mostly (Supplementary Fig. 8), showing that the highest amount of OMV protein is present in the heart at 3 h, with less present at later time points (Fig. 5B). LPS was also detected in the heart at 3 h, but the amount is less than in OMVs group (Supplementary Fig. 9). As shown in Fig. 5C,D, the activity of lactate dehydrogenase (LDH) and cardiac troponin T levels, which have been extensively recognized as parameters for the diagnosis of cardiac injury^17,18^, were significantly increased at 12 h in mice serum following OMVs administration, compared to PBS treated mice. In LPS treated mice, there was no significant increase in LDH or troponin T at any time point, even though the amount of LPS was much higher than the amount of LPS content in OMVs.

We then investigated whether OMVs induce pro-inflammatory cytokines in serum and heart tissue. Consistent with *in vitro* cytokines, OMV treated animals showed higher levels of TNF-α and IL-6 in both heart tissue and serum compared to LPS treated animals (Fig. 5E and Supplementary Fig. 10). Both TNF-α and IL-6 levels peaked at 3 h, and then gradually decreased until 12 h. The TNF-α levels at 12 h was not statistically different to the PBS treated group, whereas IL-6 still remained at significantly increased levels. RANTES, a chemokine known to be related to cardiac injury and inflammation^19^, was higher in OMV treated mice than LPS treated mice, with a peak at 12 h, significantly later TNF-α and IL-6 patterns (Fig. 5E and Supplementary Fig. 10). Moreover, OMV treated animals also showed increased leukocyte infiltration into the cardiac interstitum similar to LPS treated animals (Fig. 5F).

### Altered cardiac function in OMVs-treated mice

OMV treated mice displayed increased heart rate and heart wall thickness compared to PBS and LPS treated mice, whereas these parameters were not influenced by LPS alone (Table 1). All measures of systolic function, including cardiac output, were not influenced by LPS or OMVs.

**Table 1:** Cardiac data acquired 12 h after i.p. injection of PBS alone, LPS (40 μg) or OMVs (15 μg) in C57BL/6 mice (10 mice per group).

	PBS	LPS	OMVs
EDV (μL)	62.76 ± 11.33	67.37 ± 19.61	57.20 ± 14.43
ESV (μL)	23.06 ± 4.93	31.43 ± 20.39	22.98 ± 7.51
SV (μL)	36.69 ± 7.53	35.94 ± 16.83	33.42 ± 8.01
EF (%)	63.27 ± 4.81	54.27 ± 17.70	62.37 ± 11.21
FS (%)	33.88 ± 3.70	28.56 ± 10.49	33.68 ± 8.88
WT (mm)	0.72 ± 0.04	0.73 ± 0.05	0.79 ± 0.04^**,#^
HR (bpm)	259.13 ± 18.79	289.44 ± 53.44	360.40 ± 58.23^***,#^
CO (mL/min)	10.30 ± 2.22	10.78 ± 5.35	11.92 ± 3.55

Abbreviations: EDV, end diastolic volume; ESV, end systolic volume; SV, stroke volume; EF, ejection fraction; FS, fraction shortening; WT, wall thickness; HR, heart rate; CO, cardiac output. **P < 0.01; ***P < 0.001; versus PBS group. ^#^ *P* < 0.05; versus LPS group.

## Discussion

In this study, we show that OMVs derived from *E. coli*, without the presence of live bacteria, affect cardiomyocytes *in vitro* as well as the heart *in vivo*. Specifically, we demonstrate that the OMVs from a uropathogenic *E. coli* strain exert cytotoxicity and induce an inflammatory response in the cells, in addition to the induction of a pronounced cardiomyocyte dysfunction. These findings are further validated *in vivo*, where we show that OMV exposure leads to cardiac tissue inflammation and injury, associated with effects on cardiac function. These results in combination support the hypothesis that OMVs can contribute to sepsis induced cardiomyopathy (SIC) in the absence of bacteria. These data suggest that that OMVs can contribute to SIC, by directly influencing the heart.

When OMVs were isolated from a uropathogenic *E. coli* strain, they showed similar morphology and diameter as shown in previous studies^20,21^. We here, for the first time, show that bacterial free OMVs can induce direct cytotoxic effects on a cardiomyocyte cell line (Fig. 2). Importantly, the OMVs produced greater cytokine production than equivalent doses of free LPS, suggesting that OMVs carry other pro-inflammatory components that contribute to cytokine release. This is similar to the findings that OMVs from *Neisseria meningitides* induced pro-inflammatory cytokines and complement activation in monocytes with much higher potency than purified *Neisseria meningitidis* LPS^22,23^. Thus, these studies together suggest that OMVs evoke pro-inflammatory effects mediated by multiple components, beyond LPS.

Recordings of [Ca^2+^]_i_ dynamics is an established method to evaluate cardiomyocyte function *in vitro*. LPS-induced decreases of HL-1 cell [Ca^2+^]_i_ oscillation frequency and amplitude have previously been associated with impaired cardiac contractility^16^. In addition to the cytotoxic and inflammatory effects, we confirm that OMVs can, at comparatively low concentrations, directly affect [Ca^2+^]_i_ dynamics in HL-1 cells. indicating decreased cardiomyocyte function (Fig. 3). We have carefully compared the effects of OMVs on cardiomyocytes to LPS exposure, and demonstrate that the effect of OMVs on [Ca^2+^]_i_ dynamics is more potent than that of LPS. Notably, OMVs also had a prolonged effect on [Ca^2+^]_i_ oscillations compared to LPS (Fig. 3D and Supplementary Fig. 7), which could suggest that OMV-associated signaling is prolonged, probably by the OMVs being associated with the cells for a longer time, which is in line with OMVs being taken up by the cardiomyocytes. Other vesicular components beyond LPS are being identified to mediate OMV-associated inflammation^8^, thus we were interested to determine whether vesicular LPS or other components can induce the abnormal OMVs on [Ca^2+^]_i_ oscillations. Treating the OMVs with the LPS-blocking molecule polymyxin B did not influence the effect of OMVs on [Ca^2+^]_i_ oscillations, at a concentration that blocks the effect of purified LPS, arguing that other components of the OMVs are involved. It is known that OMVs carry LPS as well as other pathogen –associated molecular patterns (PAMPs) such as lipoteichoic acid, peptidoglycan and CpG-oligodeoxynucleotide, capable of activating TLR signaling, and several of these components can be involved in cardiac inflammation and contractility^24–26^.

Similar to our previous study in mice, showing that *E. coli* OMVs can induce systemic inflammation^21^, we here demonstrate that mice exposed to OMVs display an inflammatory phenotype, with decreased motion as well as macroscopic eye exudates. We have previously seen some uptake of OMVs in the heart, using whole body imaging^27^. In accordance with these findings, at early time points after OMV injection, OMV proteins could be shown to be present in the heart but the quantities are reduced at later time points (Fig. 5B). However, prolonged inflammation could be seen in the heart for at least 12 h. This suggests that when OMVs-induced cell activation is initiated, it progresses even with a little amount of OMVs remaining in the heart.

To date, most studies on cardiac injury and dysfunction in sepsis have been exploited in mice treated with chemically purified LPS^28,29^, and it should be noted that LPS is likely to exist outside of bacteria only as OMVs. Therefore, we here compared the effects of OMVs and LPS *in vivo*. Mice exposed to OMVs had increased serum levels of LDH activity (Fig. 5C) and troponin T (8.5 ± 0.8 ng/mL), as a specific marker of cardiac tissue damage (Fig. 5D). In comparison, mice treated with purified LPS from *E. coli* had no increase in LDH activity or troponin T release. Even if LPS did induce cytokines and chemokines, locally and systemically, the levels were lower than after OMV exposure (Fig. 5E). This again supports the concept that OMVs induce cardiac damage and inflammation, at least partly, together with non-LPS mediated pathways. Given that OMVs are composed of various immune modulators targeting toll-like receptor (TLR)^8^, it is not surprising that OMVs induce a more potent response than a single TLR agonist such as LPS. Others have previously shown that lipoprotein, one of the major component of OMVs, together with LPS act synergistically to induce cytokine production and lethal shock in mice^30^. Also, we have previously published data showing that OMVs-induced mortality was significantly higher with OMVs than with LPS alone, and polymyxin B failed to block the OMV-induced lethality^21^. Moreover, the inflammatory activity of OMVs has been shown to be partly maintained TLR2 and TLR4 knockout mice, representing that OMVs are regulated by both of these TLR, again arguing that both LPS and LPS-independent mechanism are important for the biological effect^31^.

Only mice in the OMVs treated group showed changes in cardiac function and morphology when echocardiography was performed, measured as increase in heart rate and heart wall thickness. It should be noted that baseline heart rate was relatively low compared to studies using mice anesthetized with isoflurane. The combination of ketamine/xylazine was chosen since isoflurane has been associated with cardioprotection such as reduced inflammatory response and improved survival in sepsis models^32^. The increased heart rate in response to OMVs treatment may appear difficult to reconcile with the reduced frequency and amplitude of [Ca^2+^]_i_ oscillations observed *in vitro*. However, alternations of [Ca^2+^]_i_ dynamics are expectedly connected to cardiac contractility and not to heart rate^16^.

We know that the OMVs induce inflammation in the heart, seen as increase in cytokines and inflammatory cells, and it is therefore possible that the increase in heart wall thickness is due to local inflammation. In this study, we could not demonstrate any change in other systolic and diastolic parameters in any of the groups, which may be explained by the fact that we have used a relatively low dose of both LPS and OMVs, in line with our animal ethics approval. Importantly, higher doses of OMVs will induce lethality^21^, which of course eliminates the possibility of studying cardiac function.

In addition to bacterial PAMPs, systemically release of multiple cytokines have been implicated in sepsis-induced cardiac dysfunction^33,34^. Our study shows that OMVs both induce increased concentrations of pro-inflammatory cytokines, such as TNF-α and IL-6, both in the systemic circulation as well as in hear tissue. Importantly, the heart was perfused before being processed for cytokine concentrations, removing the circulatory components of these cytokines, suggesting that local production in cardiomyocytes and/or local inflammatory cells contribute to the observed *in vivo* effects in the heart (Fig. 5E and Supplementary Fig. 10), although systemic inflammatory components may contribute. Interestingly, although significant amounts of OMVs proteins were detected in the heart at early timepoints, they then rapidly decreased, but heart injury and inflammation continued to progress, suggesting that secondary effects of OMVs contributes to the cardiac dysfunction. The relative importance of the systemic effect OMVs-induced cytokines from direct OMVs action remains to be further elucidated.

To date, there has been clinical application of OMVs as it was shown that meningococcal infection is associated with the presence of OMVs in blood from a septic patient^35^. Notably, OMVs from meningococcus are being used as vaccine, commercialized as Bexsero® (Novartis)^36^. Although bacteria themselves are generally regarded as important causes of infection and the associated inflammatory host response, it is crucial to consider that OMVs can be released from the site of infection, and travel systemically. In addition, OMVs have the capacity to deliver bacterial components across host barriers such as mucus and cell membrane^37,38^. Importantly, septic shock patients only rarely have positive blood cultures, and it is reasonable to suggest that the cytokine storm that is associated with sepsis at least partly is mediated by OMVs. This is in line with our previously published study, showing that bacteria-free OMVs from *P. aeruginosa* can induce the same degree of lung inflammation compared to live bacteria^31^. In the current study, we have taken this observation to another level, showing that bacteria-free *E. coli* OMVs can directly influence cardiomyocytes, as well as the heart *in vivo*.

SIC is associated with significantly increased morbidity and mortality rate in sepsis, and is therefore of clinical importance for further studies, as this can help the scientific community to identify novel targets for therapeutic interventions. Importantly, bactericidal antibiotics can induce an immediate worsening of sepsis, known as “antibiotic induced endotoxin release”^38^. However, it is likely that this clinical state is mediated by a rapid release of OMVs from the site of inflammation, increasing the number of circulating OMVs, thus affect other organs and inflammatory cells. Further, there is no obvious positive effects of capturing LPS with polymyxin B in clinical dialysis studies^39,40^.

This is the first study demonstrating a direct effect of OMVs on cardiomyocytes, and a clear effect of OMVs inducing SIC *in vivo*, in the absence of bacteria. Further, our data strongly argues that non-LPS components in or on OMVs are important for their pathophysiological effects. Future development of sepsis management approaches should consider the strong pro-inflammatory effects that OMVs can have in sepsis.

## Methods

### Mice

Sixty-five wild-type mice of the C57BL/6 genetic background (6 weeks old) were purchased from Charles River. The mice were housed at Experimental Biomedicine (EBM) at the University of Gothenburg, Sweden. The study was approved by the local Animal Ethics Committee in Gothenburg, Sweden (permit no 89-2016, 131-2016) and conducted in adherence with institutional animal use and care guidelines.

### Cell cultures

HL-1, a contracting cardiac muscle cell line from mouse, were purchased from Merck Millipore. The cells were grown in Claycomb medium (Sigma Aldrich) supplemented with 10% fetal bovine serum, 2 mM L-glutamine, 100 µM norepinephrine (Sigma Aldrich), 100 U/mL penicillin and 100 µg/mL streptomycin on fibronectin (BD Biosciences) pre-coated T75 flask (Thermo Fisher Scientific).

### Bacterial strains and reagents

A uropathogenic *Escherichia coli* strain was acquired to produce and isolate OMVs (bacteria were identified in a clinical microbiology laboratory with matrix-assisted laser desorption/ionization time-of-flight^41^). LPS from *E. coli* and polymyxin B were purchased from Sigma Aldrich.

### Preparation of OMVs derived from *E. coli*

*E. coli* OMVs were purified using the method described previously with modification^20^. Bacterial cultures were pelleted at 6,000 × g, 4 °C for 20 min, twice, and then the supernatant fraction was filtered through a 0.45-μm vacuum filter and was concentrated by ultrafiltration Vivaflow 200 module (Sartorius) with a 100 kDa cut-off membrane. The retentate was again filtered through a 0.22-μm vacuum filter to remove any remaining cells. The resulting filtrate was subjected to ultracentrifugation at 150,000 × g, 4 °C for 3 h and resuspended with PBS.

### TEM

OMVs from three different isolations were investigated by negative stain electron microscopy. A drop of OMVs (10 µL) dissolved in PBS, was adsorbed for 5 min onto glow-discharged 200-mesh, formvar/carbon Cu copper grids (Electron Microscopy Sciences). Then OMVs were washed two times in H_2_O and then fixed using 2.5% glutaraldehyde dissolved PBS. After two further washes in filtered water, the samples were stained using 2% uranyl acetate for 1.5 min. Negative-stained samples were examined on a digitized LEO 912AB Omega electron microscope (Carl Zeiss SMT) at 120 kV with a Veleta CCD camera (Olympus-SiS). All OMVs contained within the images were measured using the IMOD program. The vesicle size measurement was done across OMVs longest axis. In total 1189 OMVs were identified and their size measured.

### Nanoparticle tracking analysis

OMVs (5 μg/mL) were dispersed in PBS, and then the OMVs particle concentration were assessed by ZetaView analyzer (Particle Metrix GmbH). Measurements were assessed in triplicates and each individual data was obtained from two stationary layers with five times measurements in each layer. Sensitivity of camera was configured at 70 in all measurements. Data were analyzed using ZetaView analysis software version 8.2.30.1.

### LPS extraction, quantification, and silver staining

LPS were extracted from OMVs (10 µg) using the LPS extraction kit (Alpha Diagnostic International) according to manufacturer’s instructions. LPS concentrations were determined with Pierce LAL Chromogenic Endotoxin Quantitation Kit (Thermo Fisher Scientific) following manufacturer’s instructions. Silver staining of LPS was done using the silver staining kit (Thermo Fisher Scientific) according to manufacturer’s instruction.

### MTT (3-[4,5-Dimethylthiazol-2-yl]-2,5-diphenyltetrasolium bromide) Assay

HL-1 cells were seeded in a 96-well plate (2 × 10^4^ cells per well) and treated with hydrogen peroxide (500 µM), LPS (0.1 and 0.6 µg/mL) or OMVs (0.1, 1 and 10 µg/mL) for 48 h. MTT (Sigma Aldrich) was added to the cells, followed by incubation with dimethyl sulfoxide to solubilize the formazan crystal properly. Excitation was measured at 570 nm with a correction at 630 nm using a microtiter plate reader.

### ROS assay

HL-1 cells were seeded at a density of 2 × 10^4^ cells per well in 96-well plate. ROS were detected by 2′,7′-dichlorofluorescein di-acetate (10 µM; Sigma Aldrich). The probe was incubated 1 h before treatments with LPS (0.6 µg/mL) or OMVs (1 and 10 µg/mL), and fluorescence was read at 560 nm.

### Cytokine release by HL-1 and RAW 264.7 cells

HL-1 and RAW 264.7 cells were seeded in a 24-well plate (2 × 10^5^ cells per well). Hydrogen peroxide (500 µM), LPS (0.1 and 0.6 µg/mL) or OMVs (0.1, 1 and 10 µg/mL) were added to the cells, and the culture supernatant concentrations of TNF-α and IL-6 at 24 h later were measured by DuoSet ELISA Development kit (R&D Systems).

### Bacterial whole-cell lysates, periplasm and outer membrane preparation

*E. coli* whole-cell lysates were prepared using B-PER^TM^ bacterial protein extraction kit (Thermo Fisher Scientific) according to manufacturer’s instruction. Periplasm and outer membrane were purified as described previously^42^. Briefly, spheroplasts were made from bacteria by lysozyme/EDTA treatment, and periplasmic proteins were prepared from the supernatants. The spheroplasts were sonicated and pelleted at 40,000 × g for 1 h for outer membrane preparation. The outer membrane was pelleted at 40,000 × g for 90 min after incubation in 0.5% sarkosyl (Sigma Aldrich).

### Western blot analysis

HL-1 cells were treated with LPS (1 µg/mL) or OMVs (0.1 µg/mL) for 5 min. Whole-cell lysates (30 µg) were separated by 10% SDS-PAGE and transferred to a polyvinylidene difluoride membrane. The blocked membrane was then incubated with anti-beta-actin antibody (Sigma Aldrich) or anti-phospho-p65 (serine 536; Cell Signaling Technology). After incubation with horseradish peroxidase-conjugated secondary antibody, the immunoreactive bands were visualized with a chemiluminescent substrate. For Western blot using anti-OMVs antibody which was custom-made and produced using whole OMVs supplied by the authors, bacterial whole cell lysate, outer membrane proteins, periplasmic proteins, and OMVs were separated by 10% SDS-PAGE and transferred to a polyvinylidene difluoride membrane. The blocked membrane was then incubated with anti-OMVs antibody, followed by visualizing with a chemiluminescent substrate.

### Intracellular Ca^2+^ measurements

Measurements were assessed as previously described^43^, with a slight modification. Briefly, HL-1 cells were seeded on glass Petri dishes (MatTek Corporation). Cells were treated for 30 min at room temperature with 2 µL fura-2 stock solution (1 µg/µL; Thermo Fisher Scientific) added to 1 mL extracellular solution composed of 140 mM NaCl, 3.6 mM KCl, 2.6 mM CaCl_2_, 0.5 mM MgSO_4_, 0.5 mM NaH_2_PO_4_, 2 mM NaHCO_3_, 5 mM HEPES, 5 mM glucose (pH 7.4 with NaOH). During the experiments, cells were constantly perfused with the extracellular solution, and LPS (1, 5, and 10 µg/mL) or OMVs (0.5, 1, and 2 µg/mL) were added for 15 min through the perfusion system to the dish. [Ca^2+^]_i_ were monitored using a Nikon Eclipse *Ti* inverted microscope (Nikon) and a Lambda DG-4 xenon lamp illumination system (Sutter Instrument Company). Images were obtained using a QuantEM 512SC CCD camera (Photometrics) and Metafluor software (Meta imaging series 7.5; Molecular Devices). Cells were excited at 340 and 380 nm repetitively and emission light was collected above 510 nm. The fluorescence ratio (F340/F380) was calculated and the [Ca^2+^]_i_ was determined using [Equations 5]^44^ and a dissociation constant (Kd) of 224 nM. Functional regions of interest (ROIs) were regions of well synchronized cells and seven or eight regions were selected for each recording. Results were analysed using Origin Pro (OriginLab Corporation).

### Uptake of OMVs by HL-1

OMVs were stained with 5 μM of DiO (Molecular Probes) for 20 min at 37 °C. To observe the cellular uptake of OMVs, DiO-labeled OMVs were treated to Cellmask Deep Red (Thermo Fisher Scientific)-labeled HL-1 grown on coverslips and incubated for 6 h. The cells were fixed with 4% paraformaldehyde, permeabilized with 0.2% Triton X-100, and then mounted with Prolong Gold antifade reagent (Thermo Fisher Scientific). A fluorescence light microscope (Zeiss Axio observer; Carl Zeiss) was used for analysis. For the endocytosis inhibitor treatment, HL-1 cells were pretreated with 150 µM dynaosre (Sigma Aldrich) for 1 h at 37 °C, and then incubated with DiO-OMVs for 6 h as described above. Flow cytometric analysis was performed using BD FACSVerse Flow Cytometer running BD FACSuit Software (BD Biosciences) and analyzed with FlowJo Software (Tree Star Inc.).

### *In vivo* assessment of cardiac injury and inflammation

Five mice from each group were i.p injected with PBS alone, LPS (40 µg) or OMVs (15 µg) as previously described^27^, and then sacrificed at 3, 6, and, 12 h following anesthetization with a i.p bolus injection of xylazine chloride (10 mg/kg; Bayer) and ketamine hydrochloride (100 mg/kg; Pfizer AB). Blood was obtained by cardiac puncture, followed by centrifugation. The collected serum from supernatants were stored at −80 °C. The concentrations of cytokines in serum were measured using DuoSet ELISA Development kit (R&D Systems). Lactate dehydrogenase and cardiac troponin T from serum were measured by Lactate Dehydrogenase Activity Assay Kit (Sigma Aldrich) and Cardiac Troponin T ELISA Kit (MyBioSource), respectively, according to manufacturer’s instruction. To get heart whole cell lysates, heart tissue in RIPA buffer was homogenized using a gentleMACS dissociator with M tubes (Miltenyi Biotec). The cytokines, OMVs proteins, and LPS in lysates were measured using DuoSet ELISA Development kit (R&D Systems), anti-OMVs antibody (Thermo Fisher Scientific), and anti-*E. coli* LPS antibody (Abcam), respectively. For heart histology, cardiac tissue was collected and embedded in optimum cutting temperature compound (Sakura Finetek). Cryosections of 4 µm thickness were stained with hematoxylin and eosin.

### Echocardiography

The left ventricle (LV) wall thickness (WT) and systolic function were examined using transthoracic ultrasound technique as described previously^45^. Briefly, thirty mice were randomized to three groups (eight animals per group): PBS only, LPS (40 µg), and OMVs (15 µg). Mice were fully anesthetized with i.p injection once with xylazine chloride (10 mg/kg; Bayer) and ketamine hydrochloride (100 mg/kg; Pfizer AB). Body temperature was maintained as the mice were kept on a controlled warm surface during analysis. An ultrasound biomicroscope (Vevo 770 System, Visualsonics) with a high-resolution ultrasound probe was used for imaging. A LV long axis parasternal B-mode, a LV short-axis (SAX) B-MODE sequences and a motion-mode (M-MODE) were captured. LV wall thickness was calculated as the average of the anterior and the posterior wall thickness from the SAX MMODE. Fractional shortening (FS), ejection fraction (EF), stroke volume (SV), heart rate (HR), end diastolic volume (EDV), end systolic volume (ESV) were all measured from the SAX MMODE off-line with a software (Vevo 770 V3.0.0). All measurements were done in the averaged, calculated from three consecutive cardiac cycles and the animal genotype blinded to the sonographer. Cardiac output was calculated as HR * SV. FS(%) was derived using the formular 100 * [(LVIDd − LVIDs)/LVIDd] where LVIDd and LVIDs = LV internal dimensions at diastole/systole. EF(%) was calculated by using the formular 100 * [(LVIDd^3^ − LVIDs^3^)/LVIDd^3^].

### Statistical analysis

Values of results were expressed as mean and standard error of the mean (SEM). One-way ANOVA was used followed by comparison between groups using the Student’s t-test. Values were logged to meet ANOVA assumptions.

### Data Availability

The datasets generated during and/or analyzed during the current study are available from the corresponding author on reasonable request.

## Electronic supplementary material

Supplementary Information
