# Supplementary material for: Escherichia coli outer membrane vesicles can contribute to sepsis induced cardiac dysfunction
Source: Sci Rep. 2017 Dec 12;7:17434. doi: 10.1038/s41598-017-16363-9 (PMC5727113; doi:10.1038/s41598-017-16363-9)
Supplement: Supplementary file 1 — Supplementary Information [file 41598_2017_16363_MOESM1_ESM.pdf]

Escherichia coli outer membrane vesicles can contribute to sepsis induced cardiac dysfunction

Kristina Svennerholm[1,#], Kyong-Su Park[2,#], Johannes Wikström[3], Cecilia Lässer[2], Rossella Crescitelli[2], Ganesh V. Shelke[2], Su Chul Jang[2,4], Shintaro Suzuki[2], Elga Bandeira[2], Charlotta S. Olofsson[5], Jan Lötvall[2,4,*]

[1]*Department of anesthesiology and Intensive Care Medicine, Institute of Clinical Science, Sahlgrenska Academy, University of Gothenburg, Gothenburg 40530, Sweden*

[2]*Krefting Research Centre, Institute of Medicine, University of Gothenburg, Gothenburg 40530, Sweden*

[3]*Cardiovascular & Metabolic Disease Innovative Medicines, AstraZeneca R&D, Mölndal 43150, Sweden*

[4]*Codiak BioSciences Inc, 500 Technology Square, 9th floor, Cambridge, MA 02139, USA*

[5]*Department of Physiology/Metabolic Physiology, Institute of Neuroscience and Physiology, Sahlgrenska Academy, University of Gothenburg, Gothenburg 40530, Sweden*

#These authors contributed equally to this work

*Correspondence and requests for material should be addressed to J.L (email: jan.lotvall@gu.se)

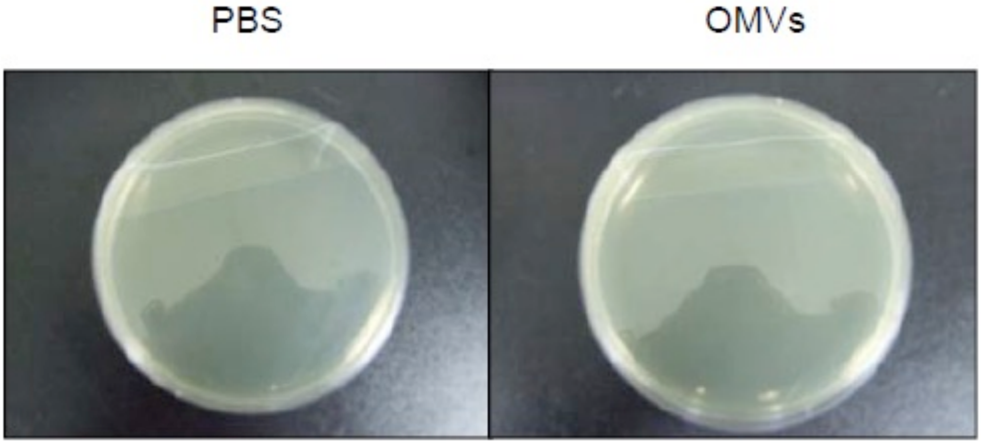

Supplementary Fig. 1. No contamination of bacteria in OMVs preparation. PBS or purified OMVs (10 μg) were spread on Luria-Bertani plates, followed by incubating at 37˚C for 24 h.

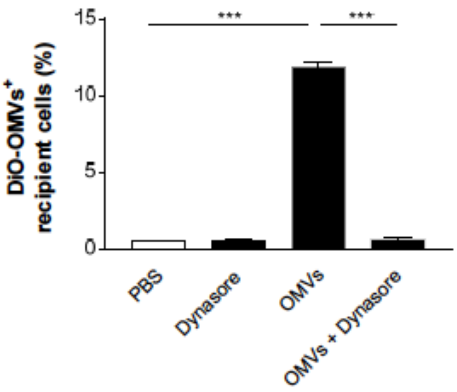

Supplementary Fig. 2. The effect of dynosore on the uptake of OMVs into HL-1 cells. HL-1 cells were pretreated with dynosore for 1 h at 37˚C, and then incubated with DiO-OMVs for 6 h at 37˚C. The uptake of the fluorescently labelled OMVs by cells was detected with flow cytometry, and data represent the percentage of DiO-positive cells of two independent experiments. ***, $P<0.001$; Error bars indicate SEM.

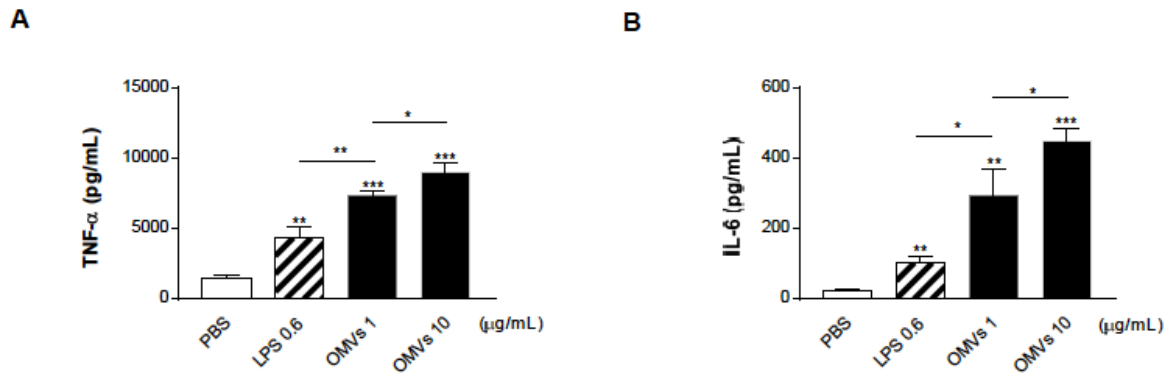

Supplementary Fig. 3. OMVs activate macrophage to provoke cytokine production. LPS (0.6 μg/mL) or OMVs (1, 10 μg/mL) were added to RAW 264.7 cells, and the culture supernatant concentrations of TNF-α and IL-6 24 h later are shown in panel **A** and **B**, respectively. *, $P<0.05$; **, $P<0.01$; ***, $P<0.001$; ns, not significant; versus PBS group. LPS (0.6 μg/mL) and OMVs (10 μg/mL) group were also compared with OMVs (1 μg/mL) group. Error bars indicate SEM. n=3.

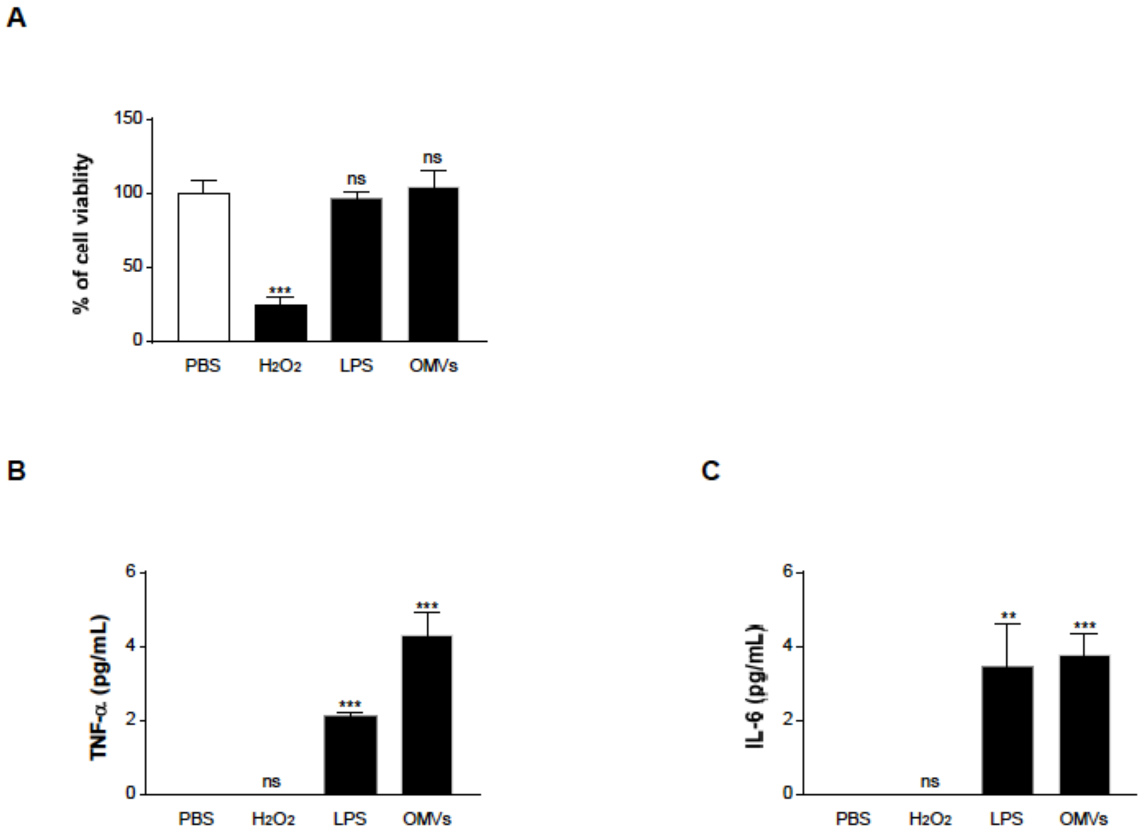

Supplementary Fig. 4. The effect of H_2O_2 (hydrogen peroxide) and lower concentration of LPS and OMVs on viability and cytokine production in HL-1 cells. (**A**) Cells were exposed to H_2O_2 (500 μM), LPS (0.1 μg/mL) or OMVs (0.1 μg/mL) for 48 h and cell viability was assessed by MTT assay. Results are expressed in percentage of control. (**B** and **C**) H_2O_2 (500 μM), LPS (0.1 μg/mL) or OMVs (0.1 μg/mL) were added to HL-1 cells, and the culture supernatant concentrations of TNF-α and IL-6 24 h later are shown in panel **B** and **C**, respectively. **, $P<0.01$; ***, $P<0.001$; ns, not significant; versus PBS group. Error bars indicate SEM. n=3.

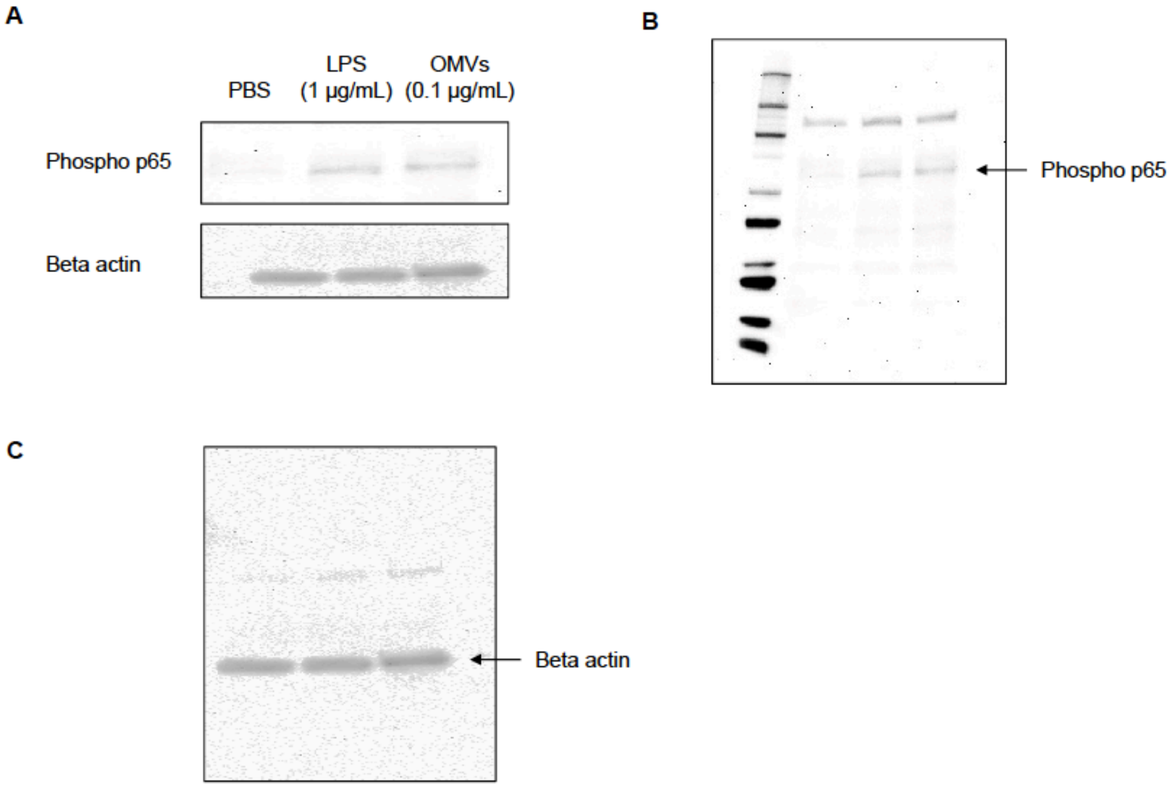

Supplementary Fig. 5. OMVs induce the phosphorylation of p65 in HL-1 cells. (**A**) Cells were treated with LPS (1 µg/mL) or OMVs (0.1 µg/mL) for 5 min. The whole-cell extracts were prepared as described in Methods. 40 µg of extracts were probed with anti-phospho-p65 (serine 536) antibody (1/1000 dilution). For loading control, the blots were stripped and reprobed with anti-beta-actin antibody (1/1000). The images were cropped from different parts of the same gel. (**B** and **C**) Additional raw images of the Western blots with anti-phosphos-p65 antibody (**B**) and anti-beta-actin antibody (**C**) shown in Supplementary Fig. 5a. From left to right: PBS group, LPS (1 µg/mL) group, OMVS (0.1 µg/mL) group.

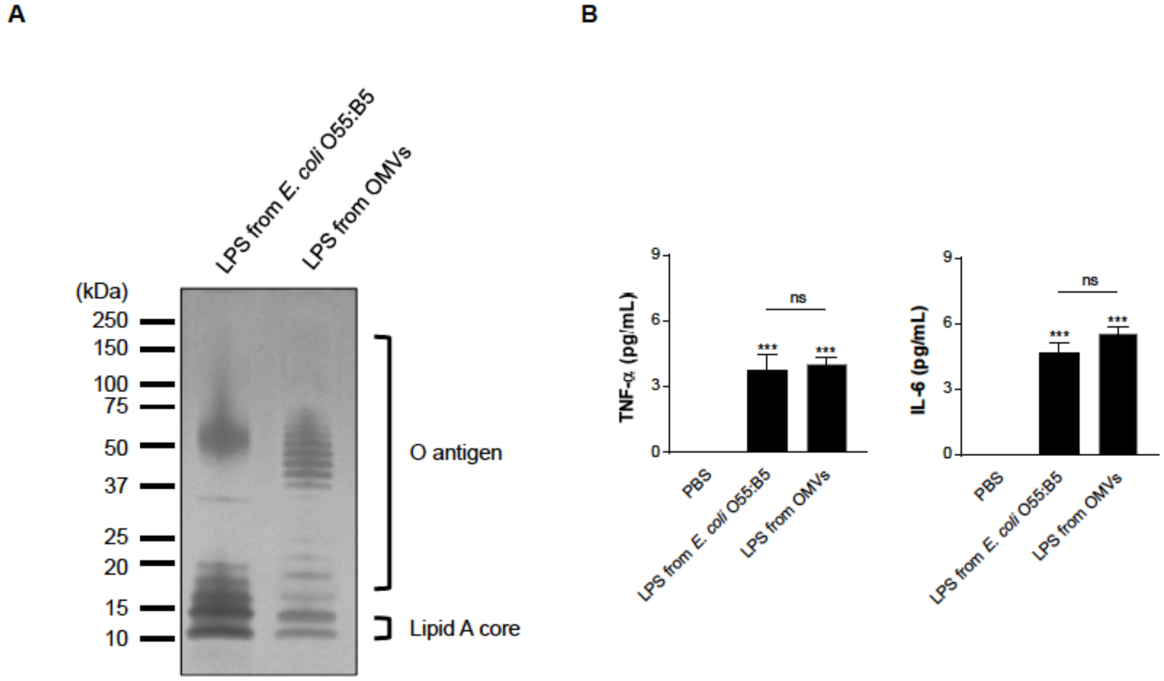

Supplementary Fig. 6. The molecular and inflammatory activity comparison between LPS purchased and LPS from OMVs. (**A**) Both LPS (10 μg) were subjected to SDS-PAGE analysis and visualized by silver staining. This is the whole image of SDS-PAGE gel. (**B**) Both LPS (1 μg/mL) were added to HL-1 cells, and the culture supernatant concentrations of TNF-α and IL-6 24 h later were measured. ***, $P<0.001$; ns, not significant; versus PBS group. LPS from *E. coli* O55:B5 group was also compared with LPS from OMVs group. Error bars indicate SEM. n=3.

A

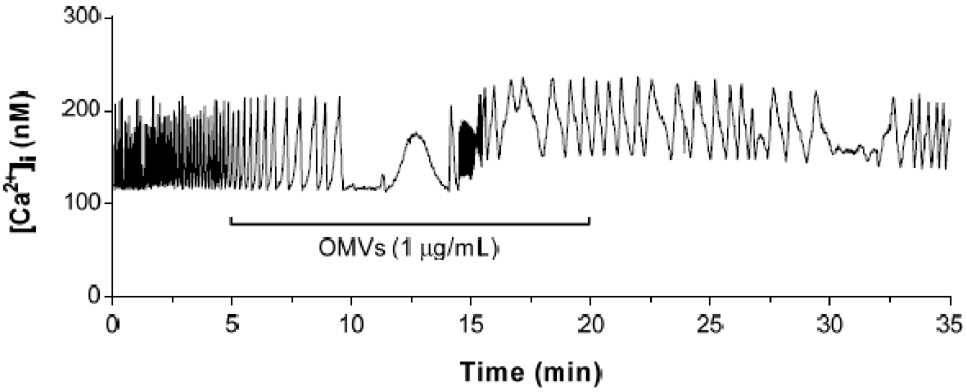

B

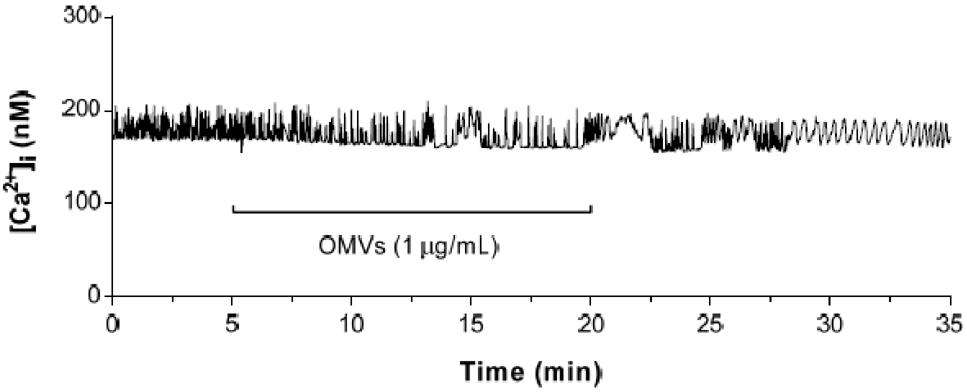

Supplementary Fig. 7. OMVs induce changes in intracellular calcium. OMVs (1 µg/mL) was applied for 15 min to the cells which were continuously perfused with the extracellular solution. Horizontal lines show application time of OMVs.

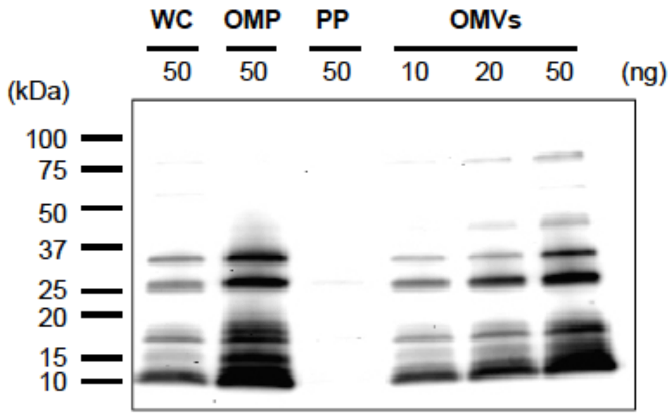

Supplementary Fig. 8. Western blot analysis of bacterial whole-cell lysates (WC), outer membrane proteins (OMP), periplasmic proteins (PP), and OMVs with anti-OMVs antibody. This is the whole image of the Western blots.

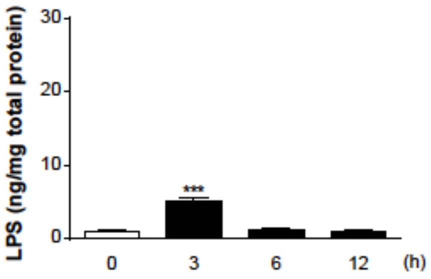

Supplementary Fig. 9. LPS was delivered to the heart tissue at early time point. Heart lysates were prepared from mice injected with LPS as shown in Fig. 5a, followed by measurement of LPS using anti-*E. coli* LPS antibody. ***, *P*<0.001; versus 0 h group. Error bars indicate SEM. n=5.

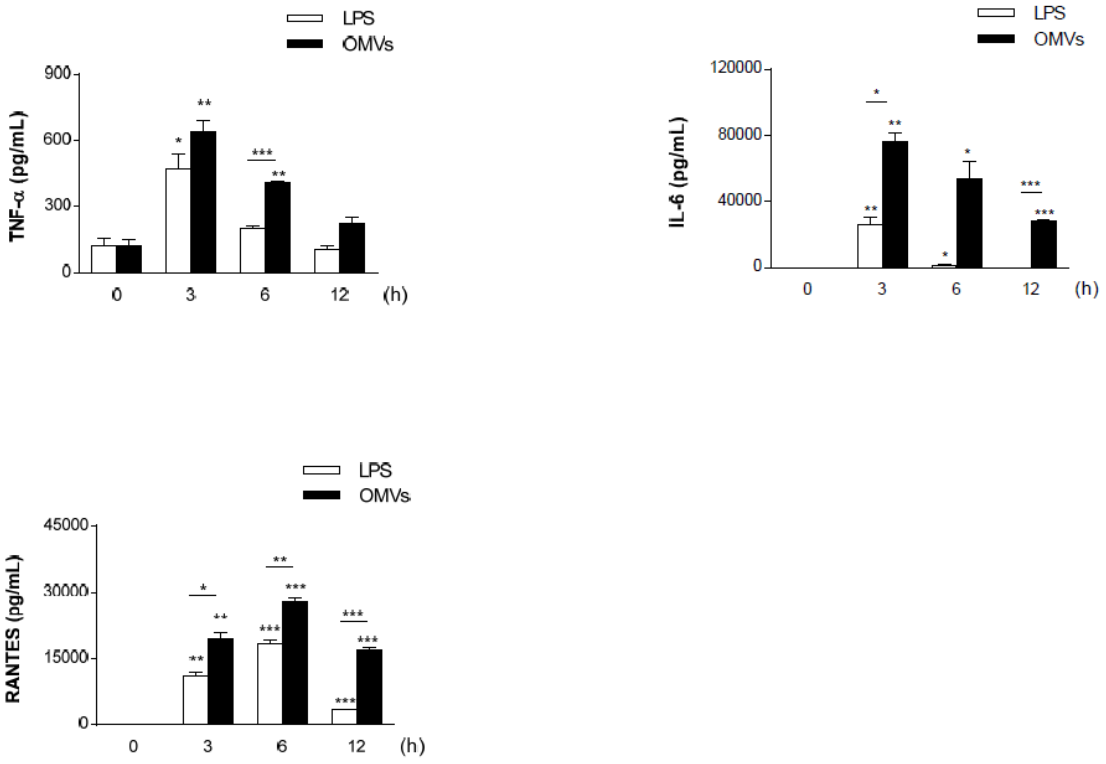

Supplementary Fig. 10. OMVs increase TNF-α, IL-6, and RANTES in serum. Serum were prepared from mice injected with OMVs or LPS as shown in Fig. 5a, followed by measurement of TNF-α, IL-6, and RANTES by ELISA. *, *P*<0.05; **, *P*<0.01; ***, *P*<0.001; versus 0 h group. Error bars indicate SEM. n=5.
